# Case management via telephone counseling and SMS for weight maintenance in adolescent obesity: study concept of the TeAM program

**DOI:** 10.1186/2052-9538-1-8

**Published:** 2014-05-29

**Authors:** Jana Markert, Sabine Herget, Stefanie Marschke, Thomas Lehnert, Christian Falkenberg, Susann Blüher

**Affiliations:** Leipzig University Medical Center, IFB Adiposity Diseases, University of Leipzig, Philipp-Rosenthal-Str. 27, Leipzig, D-04103 Germany; Department for Medical Sociology and Health Economics, Hamburg Center for Health Economics (HCHE), University Medical Center Hamburg-Eppendorf, Martinistr. 52, Hamburg, D-20246 Germany; German statutory pension insurance, Department North, Medical Rehabilitation Hospital “Satteldüne”, Tanenwai 32, Nebel/Amrum, D-25946 Germany

**Keywords:** Obesity, Adolescence, Weight maintenance, Telephone counseling, Tailored short messages, Systemic counseling, Case management, TeAM program, Rehabilitation research

## Abstract

**Background:**

In-patient obesity treatment programs for adolescents are associated with good success and substantial weight loss. However, maintaining weight loss remains a challenge. This article presents the concept of the *TeAM* (*Te*lephone counseling as *A*diposity *M*anagement) program. TeAM is an innovative, weight maintenance program for obese adolescents after in-patient therapy. It applies the case management approach in combination with new media (telephone counseling, web forum, and SMS messaging). Adolescents (14–18 years) were recruited via German rehabilitation hospitals. The intervention of the TeAM program consists of telephone counseling through trained case managers in order to maintain body weight reduction (expressed as BMI-SDS: body mass index standard deviation score) achieved during an in-patient obesity therapy. At baseline and after completion of the program, participants provide anthropometric measures (obtained by trained medical staff) as well as information on socio-demographics, usage of health services, psychosocial status, daily physical activity, media consumption, and eating behavior. The core of the intervention is regular telephone contact with the adolescent participants combined with tailored SMS messages. Telephone counseling is based on the systemic approach and addresses the topics of mental hygiene, physical activity, sedentary behavior, diet and eating behavior.

**Results:**

Baseline data of the feasibility study: Thirty-eight adolescents were recruited for the feasibility study (14 male, 24 female; mean age 15.82 years); out of them, ten participants lived with a single parent; 68% planned to graduate from school without pre-requisites for university admission (O-level). The mean weight loss during in-patient treatment was 0.32 BMI-SDS units. Mean BMI at the start of intervention was 31.93 kg/m^2^, corresponding to a mean BMI-SDS of 2.48.

**Conclusions:**

Weight maintenance treatment programs for adolescent obesity utilizing new media are a promising approach as they reach adolescents directly within their everyday life.

**Trial registration:**

DRKS00004583.

## Background

Obesity accounts for 2-5% of total healthcare costs of industrialized countries [1, 2]. Moreover, obese individuals accrue medical costs approximately 30% higher than those of normal weight peers, as a review published in 2011 on the basis of eight different studies (conducted 1998–2009) has shown [3]. As overweight and obesity are likely to persist from childhood into adult life [4], it has to be treated effectively from early on. Drug therapy as well as bariatric surgery are no treatment options for the majority of the pediatric population, therefore lifestyle intervention remains the most well-established and recommended therapy for obese children and adolescents [5, 6]. Hence a treatment option for juvenile obesity is in- and/or out-patient obesity therapy, aiming to achieve weight loss and a sustained effect on lifestyle-related factors. However, maintaining weight loss (stabilization of BMI-SDS) after obesity therapy remains a challenge. Most interventions are marked by considerable relapse in the follow-up period [7].

While extended care has considerable impact on the long-term maintenance of weight loss [8], its medical application remains scarce, especially in pediatric medicine. Available aftercare interventions in adulthood address weight loss maintenance by extending treatment contact [9] and/ or treatment content [10]. They are mainly based on personal contacts and are therefore time and cost intensive. Digital communication such as telephone counseling, e-mail contact and internet platforms might provide low-threshold, cost effective options, which have to be explored. Preliminary data suggest that “new media” might be an effective tool to reach that age group for distinct interventions addressing health related topics [11–14]. Thus, structured weight maintenance treatment based on media communication for adolescents seems promising.

To our knowledge, no obesity intervention study for children and adolescents has to date examined the impact of telephone counseling in aftercare weight maintenance. Thus, the TeAM (*Te*lephone counseling as *A*diposity *M*anagement) program presents an innovative approach for weight maintenance in overweight and obese adolescents based on telephone counseling and applying new media, such as tailored SMS messages, following in-patient weight loss therapy. The aims of this article are a) to present the study concept for the TeAM program, and b) to present baseline data of the feasibility study.

## Methods

### Feasibility study

#### Study design and study center

Currently, a pilot study is being conducted for four months to test the feasibility of the TeAM program. Reporting of this trial is in accordance with the 2010 CONSORT Statement [15]. TeAM was registered in the German Clinical Trials Register in 2012 (DRKS00004583), available in the International Clinical Trials Registry Platform of the WHO.

In the feasibility study two distinct modes of intervention exist, i.e. there are two intervention groups and one control group. One intervention group receives telephone counseling and tailored SMS messages as reminders of lifestyle-changes. The other intervention group receives telephone counseling, tailored SMS messages and has additional access to a password-protected web-forum for interaction with other participants. The control group receives no intervention and is offered medical care as usual, based on German medical guidelines for childhood and adolescent obesity [6]. The intervention is implemented as case management approach within the home setting of the participants. This supports the low-threshold approach recommended in the treatment of (pediatric) obesity [6].

The intervention is conducted at the Integrated Research and Treatment Center (German acronym IFB) AdiposityDiseases at the Faculty of Medicine at the University of Leipzig. The data centre of the IFB is located at the Clinical Trial Center of the University of Leipzig (German acronym KSL); it serves as an independent facility of the Faculty of Medicine at the University of Leipzig. All trial-related processes follow the standard operation procedures (SOPs) of the KSL, which has been audited externally. The KSL supports data analysis of the study.

Multicentered recruitment is performed at six rehabilitation hospitals within Germany. Clinical baseline assessments are conducted within these six cooperating hospitals. The post intervention assessments are performed by the local pediatrician or local general practitioner of the participants.

CrescNet is an auxological collaborative network connecting German pediatricians in practice with endocrine specialists nationwide. One of its main objectives is to collect in a standardized manner data on anthropometry [16]. It serves as study database and supports the documentation of the entire intervention process.

#### Participant eligibility

Adolescents aged 14–18 years who completed a structured in-patient obesity therapy program (four to six weeks of in-patient treatment) with the main indication of pediatric obesity [17] were eligible for study participation. Written informed consent of parents or guardians and of adolescents themselves was obtained prior to the intervention. Adolescents with current involvement in weight loss treatment, with psychiatric conditions interfering with participation (e.g. eating disorder, psychosis), with medication interfering with participation or weight maintenance, and/ or underlying chronic disease interfering with weight maintenance were excluded from the trial.

#### Recruitment and allocation to condition

Screening for eligible adolescents and recruitment of study participants were performed directly via the collaborating German rehabilitation hospitals. The intervention started within a period of six weeks after completion of an in-patient obesity therapy.

Adolescents were randomized to one of the intervention groups or to the control group (observational group). The participants completed a structured in-patient obesity therapy and are thus aware of their medical problem. They agreed to participate in a weight maintenance intervention and filled out the baseline study questionnaire. Randomization was carried out centrally using pre-prepared lists stratified by gender. Participants were assigned in the order in which they returned baseline study material. The staff at the randomization center had no influence on this order and did not select participants. Hence, this procedure was free of bias. Thus, the difference between both groups reflects the impact of the intervention, free of bias induced by awareness of the problem of weight maintenance or willingness to participate.

#### Intervention goals

The goal of the TeAM-program is to transfer effects of in-patient treatment to everyday life by improving intrinsic motivation of participants and by facilitating the development of weight maintenance strategies. In the feasibility study, the percentage of adolescents who agreed to participate and who adhere to the intervention are explored. Additionally, the study reveals the most suitable and accepted mode and design of an aftercare treatment for adolescents in order to maintain weight loss.

#### Intervention methodology

The telephone interviews are based on an interviewing technique modeled on the systemic approach by Steve De Shazer [18]. This solution-focused brief counseling is performed by trained case managers according to a standardized manual. One participant is accompanied by the same person during all intervention stages (case management approach). To improve the compliance of the participants, written informed consent is required from parents and adolescents themselves. Additionally, within the first counseling session an oral contract about rules of reliability and discretion is made between participant and case manager. This process is documented in the central study database (CrescNet). To ensure the quality of the counseling and adherence to the study manual, external supervision is performed regularly.

##### Session structure

The study manual describes each counseling session in detail. Generally, the sessions consist of an introduction, a counseling part, a goal setting part and a closing part. The introduction serves to welcome and motivate the participant, and to provide feedback later on in the intervention process. The counseling part is adapted to the session theme (see also Table 1). A goal setting part helps the participant to formulate up to two concrete minor tasks, which should be implemented in the participants’ everyday life until the next session. The closing part serves to summarize results of the session and to motivate the participant to work on his/her goals. The documentation of the telephone sessions is implemented in the CrescNet database in a standardized manner.

**Table 1:** **Detailed intervention plan for an individual participant**

Timeline [weeks]	End of in-patient treatment	6	7	8	9	10	11	12	13/14	15	16/17	18
Telephone counseling		Enrolment session + parents*		Anamnestic session		Basic session mental hygiene		Basic session physical activity		Basic session nutrition		Final summary + parents*
SMS		Appointment reminder	Task reminder	Appointment reminder	Task reminder	Appointment reminder	Task reminder	Appointment reminder	Task reminder	Appointment reminder	Task reminder	Appointment reminder
Study visits and online questionnaires	Baseline assessment											Post intervention assessment

*The respective counseling session is hold with participants and their parents.

##### Session content

Each counseling interview takes approximately 30 minutes (the anamnestic session takes 45 minutes) and addresses the adolescents directly. The telephone interviews contain the following aspects: Enrolment session to inform the family about the upcoming intervention and to assess socio-demographic parameters of the parents. Anamnestic session: What has been reached to date? What are the goals for the telephone counseling? Basic sessions (three consultations): What has been reached regarding health behavior? Is there still support needed for certain areas? Final session: Summary of all information, maintaining long-lasting change of distinct lifestyle habits, and program evaluation. Feedback of counseling contents is given to parents after authorization by the participant.

The case manager monitors the goal setting in accordance with the guideline on diagnostics, treatment, and prevention of obesity in childhood and adolescents [6] of the working group pediatric obesity (German acronym AGA) of the German Obesity Society (German acronym DAG).

##### Tailored short text messages (SMS)

At the end of each counseling session the participant develops up to two (minor) individual tasks to work on until the next telephone contact. These action planning tasks are in essence homework in order to integrate obesity preventing behavior into everyday life. The fulfillment of the task is evaluated between participant and case manager at the beginning of the following session.

To remind the participant, tailored short messages (SMS) are sent to the participants’ mobile phone. Tailoring is performed according to the self-composed task of the participant. The SMS consists of a short welcome part, a motivation and a reminder, and is sent by the respective case manager.

Example one: Hello [participants’ name], hopefully you have had a nice day! Your idea to use the stairs instead the elevator is great. Please keep this in mind. I look forward to our next phone call. All the best [case manager’s name].

Example two: Dear [participants’ name], our last talk was great. It’s fantastic how you manage difficulties and find solutions by yourself. Good luck in exploring slow eating. Using cutlery can be helpful. Cheers [case manager’s name].

Improvement of adherence by use of text messaging has been found in several intervention studies [19–22]. Experience of former telephone counseling projects within our group [23], Markert J*, Herget S*, Petroff D, Gausche R, Grimm A, Kiess W, Blüher S: Telephone-based adiposity prevention for families with overweight children (T.A.F.F.-study): 1 year outcome of a randomized, controlled trial / submitted] also indicates the utility of systematic appointment reminders one day ahead of a counseling date to encourage participants’ attendance at their counseling session. Reminders are generated by an internet-based SMS platform. During the intervention, a confrontation-technique is used to rebuild motivation and scrutinize participants’ compliance. To support adherence and to provide public information, the study runs an official facebook-page (one way communication).

#### Frequency and scope of study visits

Study visits in the feasibility study include one baseline visit after written agreement and one closing visit after the end of intervention. All anthropometric measurements are performed by trained staff according to standardized procedures, as self-reported data on body weight have been shown to be biased [24].

#### Study endpoints/ outcome measures

In the feasibility study, the acceptance of and adherence to the aftercare treatment approach will be evaluated. Acceptance is defined ≥ 10% of adolescents (14–18 years) receiving in-patient obesity treatment in a cooperating rehabilitation hospital, declare their informed consent into study participation. The cut off point for adherence is 33.3%, which means the feasibility study will be successful if fewer than 33.3% of randomized adolescents drop out of the intervention. Additionally, study questionnaires consisting of the scales planned to be analyzed within the efficacy study (RCT) are administered, in order to explore the feasibility of online study questionnaires and its integration in the study data base.

#### Data analysis

Standard methods of descriptive analysis were applied on the baseline data of the feasibility study using R version 2.15.0 [25].

### Envisaged design and analyses of the planned efficacy study (RCT)

If the TeAM-program proves to be feasible, an randomized controlled trial (RCT) with six months of intervention will be conducted in order to evaluate the efficacy of the TeAM-program.

Inclusion and exclusion criteria, recruitment, intervention goals, and intervention methods of the RCT are planned to be equal to the ones of the feasibility study. In the RCT only one intervention group receiving the mode of intervention superior over the other, as shown by the feasibility study, is planned.

The primary outcome of this efficacy study will be the comparison of the changes in BMI-SDS from randomization to six months follow-up between the intervention group and the control group. In the calculation of SDS values, age and sex are taken into account. The primary endpoint will be evaluated by analysis of covariance, using BMI-SDS at follow-up as dependent variable, change in BMI-SDS during in-patient treatment, BMI-SDS at baseline, age, sex and the assigned prevention manager as covariates, and the randomized group as factor. All subjects with BMI-SDS available at baseline and follow-up will be included on the intent-to-treat basis. Missing values will be imputed, making conservative assumptions regarding the success of drop-outs. Quantitative secondary endpoints (including BMI, waist- and hip-circumferences, WHR, blood pressure, skinfolds, psychosocial well-being and health-associated quality of life, daily physical exercise, daily sedentary behavior, composition of dietary intake, eating behavior) will be evaluated by analyses of covariance.

Estimates for the change in BMI-SDS were be made from a randomized, controlled trial on maintaining weight loss with two competing interventional programs published in 2007 [26] after taking into account the sample size and based on the assumption that the error bars for the changes from their baseline (before initial weight loss) roughly correspond to those for the BMI-SDS changes in our study. The above literature suggests that the BMI-SDS of the intervention group will decrease by about 0.05 ± 0.20 in six months and the control group will increase by 0.04 ± 0.20 in that same period of time. This paper [26] further suggests that R2 for the covariates will be roughly 0.42. A power analysis for ANCOVA using these parameters shows that 46 subjects per arm are necessary to reach a power of 80% at a significance level of 5% (PASS 11, Version 11.0.2). We assume 22% of drop-out [26], meaning that we intend to randomize n = 118 to the trial (roughly 59 per arm).

The feasibility study serves, among other things, to help plan the sample size of the trial. Therefore, the sample size determined above may be adjusted once information from the feasibility study is available.

### Ethical approval

The entire study is carried out according to the ethical principles originating from the Declaration of Helsinki and is consistent with the guidelines about Good Clinical Practice of the International Conference on Harmonisation (ICH-GCP). The protocol of the study design has been approved by the local ethical committee of the University of Leipzig (AZ 295-12-24092012).

## Results

### Baseline data of the feasibility study

Thirty-eight adolescents were enrolled into the feasibility study and started the intervention. For thirty-five adolescents, the respective rehabilitation hospital has provided complete anthropometric and clinical parameters.

### Socio-demographic parameters

14 boys (37%) and 24 girls (63%) were included in the feasibility intervention. For allocation of study groups see Table 2. The mean age of participants was 15.82 ± 1.24 years (n = 35). One fourth of them (26%) lived with a single parent. The majority of participating adolescents (68%) aimed an O-level school certificate (graduate from school without pre-requisites for university admission).

**Table 2:** **Baseline characteristics of the participants from the feasibility study (N = 38)**

	Mean ± SD or N (%)			
	Intervention I	Intervention II	Control group	Feasibility study in total (Σ)
Number of participants	12 (31.6%)	13 (34.2%)	13 (34.2%)	38 (100%)
Number of females	8 (66.7%)	9 (67.2%)	7 (53.9%)	24 (63.2%)
Age [years]	15.26 ± 0.93	16.22 ± 1.46	15.92 ± 1.15	15.82 ± 1.24
Duration of in-patient treatment [days]*	36.09 ± 9.85	41.67 ± 8.39	31.00 ± 7.20	36.00 ± 9.39
Weight loss [BMI-SDS]*	0.35 ± 0.17	0.30 ± 0.11	0.30 ± 0.12	0.32 ± 0.13
BMI [kg/m^2^]*	30.82 ± 3.61	33.75 ± 6.14	31.11 ± 2.71	31.93 ± 4.50
BMI-SDS*	2.34 ± 0.55	2.69 ± 0.76	2.41 ± 0.38	2.48 ± 0.59
Persons with co-morbidities*	10 (28.6%)	10 (28.6%)	8 (22.9%)	28 (80.0%)
Aspired school level certificate:				
Less than O-level	2 (16.7%)	1 (07.7%)	3 (23.1%)	6 (15.8%)
O-level	8 (66.7%)	10 (76.9%)	8 (61.5%)	26 (68.4%)
A-level	2 (16.7%)	2 (15.4%)	2 (15.4%)	6 (15.8%)
Single parenthood	3 (25.0%)	5 (38.5%)	2 (15.4%)	10 (26.3%)

Data are presented as mean ± SD or as numbers (%). *Medical baseline data is available for 35 participants.

### Anthropometric and clinical parameters

Mean time of stay at the rehabilitation hospital was 36 ± 9.39 days. The mean weight loss during in-patient treatment was 0.32 points of BMI-z-score, resulting in a mean BMI of 31.93 kg/m^2^ and corresponding to a BMI-SDS of 2.48 at start of the feasibility study. Most of the participants (n = 28; 80%) showed one or more co-morbidities, such as arterial hypertension (n = 9; 26%), bronchiatic asthma (n = 6; 17%), dyslipidemia (n = 4; 11%), endocrine dysfunction, e.g. hypothyroidism or insulin resistance (n = 4; 11%), and orthopedic symptoms (n = 4; 11%). For detailed information on baseline characteristics and group differences see Table 2.

## Discussion

To the best of our knowledge, this study is the first internationally published study concept of a weight maintenance treatment approach for adolescent obesity based on telephone counseling following in-patient obesity-therapy. In Germany, validated aftercare treatment concepts for pediatric obesity are scarce to date. As obesity prevalence is still high in adolescence [27], this age group remains a target group for obesity treatment and weight maintenance approaches. Metabolic co-morbidities seem to cumulate in the pubertal age in obese subjects [28], emphasizing that a structured clinical investigation in adolescents obesity is warranted. Limited data are available regarding the effects of e-mail and/ or telephone approach for weight maintenance both in adults and adolescents [29–31]. Available studies describe a positive effect of telephone based interventions to improve physical activity and dietary behavior [32, 33]. Important factors for the long-term success of such approaches with regard to weight maintenance include timely feedback and social support, but also interaction with other participants [34] and individualized (tailored) information [35, 36] as provided within the TeAM program. In pediatric medicine the involvement of parents is recommended [37]. However, addressing adolescents via their parents has been shown to be inappropriate [23]. Therefore weight management interventions directly addressing adolescents are needed. Preliminary data suggest that new media and telephone contact might be an effective tool to reach that age group [38]. Therefore the TeAM-program uses online-questionnaires, E-Mail, an internet-forum, and mobile phones as integrated everyday life technology in adolescence. So far, aftercare approaches to sustain effects of in-patient treatment have been mainly designed for the adult population, but should also be applied in pediatric medicine. The TeAM program aims to address adolescents aged 14 to 18 years. Baseline data from the feasibility study suggests that the program seems to address adolescents in an appropriate manner. The proportion of girls reflects distribution between genders seen in in-patient obesity therapy. There exists a wide range of weight status in our program, ranging from overweight (BMI-SDS of 1.29) to extremely obesity (BMI-SDS of 4.19), according to German reference guidelines [17]. Evaluation of participation rates will be performed after all participants have finished the intervention of the feasibility study.

## Conclusion

The advantages of the present weight maintenance treatment approach could be its local and also temporal flexibility, its home-setting, low-threshold design, and its possibility for tailored counseling and direct personalized feedback (case management regime). Thus, such an approach for structured weight maintenance in adolescent obesity seems to be promising and should be investigated for its feasibility and efficacy.
